# Ventilation and Ventilators

**Published:** 1894-02-03

**Authors:** 


### PRACTICAL DEPARTMENTS.

### VENTILATION AND VENTILATORS.

JOHN KING (LIMITED), LIVERPOOL.

It cannot be said that there is any lack of diversity of apparatus wherewith to produce, or attempt to produce, that much sought after but rarely attained change of air commonly called ventilation. And of all the forms of mechanism applied to this purpose probably the one to which most attention has been directed is the cowl. Cowls of all sorts, shapes, and sizes, with revolving heads that spin round with the slightest movement of the air; with funnel-shaped heads moving only to face the prevailing wind, or with no movable parts about them; cowls ugly and cowls ornamental; cowls made apparently to do the same work on diametrically opposite principles—all can be had in abundance, and each is guaranteed to be the most perfect appliance in the market. Inasmuch as all these cowls depend for their efficiency on the well-known action of a current of air crossing an aperture in a tube or shaft at right angles, thereby producing a partial vacuum, which causes an up draught in the pipe, it can scarcely be supposed that all these varying forms of cowl do their work with equal efficiency. As a matter of fact this is not the case, and it has been proved by experiment that the movement of air through some kinds of cowl is not perceptibly greater than that through a freely open pipe. Indeed, it is obvious that however well devised a cowl may be there must be times when the movement of air is so slight as to have but a feeble effect in its action, and this notwithstanding the fact so strongly emphasised by all makers of cowls that the number of hours in a year when there is absolutely no wind is so small as to be a negligible quantity. We have before us the prospectus of a cowl called "The Tubular," invented by Mr. Barker, and made by John King (Limited), of Liverpool. It consists of a central square shaft, around which are grouped four extracting tubes, each having a vertical outlet opening with lips curved towards the central shaft. From whatever direction the wind is blowing it must enter at one or two of the openings between the four extracting tubes, and pass through the constricted passages between these tubes and the central shaft. In doing this the velocity of the wind is increased and the air in the tubes is sucked out. This produces a partial vacuum in the conical chamber above the top of the central shaft, which is at once filled with air from the latter; and thus it is claimed that a continuous up-current is maintained in the shaft. The point that strikes us as specially noteworthy in this cowl is the arrangement by which the four exhaust tubes are made independent of one another, instead of opening into one central chamber. And although we cannot altogether accept all the qualities of perfection claimed for the apparatus, we certainly think it is the most ingeniously contrived cowl we have yet seen for utilising wind power to the utmost extent possible.

